# A Garlic Derivative, S-allylcysteine (SAC), Suppresses Proliferation and Metastasis of Hepatocellular Carcinoma

**DOI:** 10.1371/journal.pone.0031655

**Published:** 2012-02-28

**Authors:** Kevin T. P. Ng, Dong Yong Guo, Qiao Cheng, Wei Geng, Chang Chun Ling, Chang Xian Li, Xiao Bing Liu, Yuen Yuen Ma, Chung Mau Lo, Ronnie T. P. Poon, Sheung Tat Fan, Kwan Man

**Affiliations:** 1 State Key Laboratory for Liver Research, Department of Surgery, LKS Faculty of Medicine, The University of Hong Kong, Hong Kong Special Administrative Regions (SAR); 2 Department of Anesthesiology, Cancer Institute, Tianjin Medical University, Cancer Hospital, Tianjin, China; The University of Hong Kong, China

## Abstract

**Background:**

Hepatocellular carcinoma (HCC) is highly malignant and metastatic. Currently, there is no effective chemotherapy for patients with advanced HCC leading to an urgent need to seek for novel therapeutic options. We aimed to investigate the effect of a garlic derivative, S-allylcysteine (SAC), on the proliferation and metastasis of HCC.

**Methodology/Principal Findings:**

A series of *in vitro* experiments including MTT, colony-forming, wound-healing, invasion, apoptosis and cell cycle assays were performed to examine the anti-proliferative and anti-metastatic effects of SAC on a metastatic HCC cell line MHCC97L. The therapeutic values of SAC single and combined with cisplatin treatments were examined in an *in vivo* orthotopic xenograft liver tumor model. The result showed that the proliferation rate and colony-forming abilities of MHCC97L cells were suppressed by SAC together with significant suppression of the expressions of proliferation markers, Ki-67 and proliferating cell nuclear antigen (PCNA). Moreover, SAC hindered the migration and invasion of MHCC97L cells corresponding with up-regulation of E-cadherin and down-regulation of VEGF. Furthermore, SAC significantly induced apoptosis and necrosis of MHCC97L cells through suppressing Bcl-xL and Bcl-2 as well as activating caspase-3 and caspase-9. In addition, SAC could significantly induce the S phase arrest of MHCC97L cells together with down-regulation of cdc25c, cdc2 and cyclin B1. *In vivo* xenograft liver tumor model demonstrated that SAC single or combined with cisplatin treatment inhibited the progression and metastasis of HCC tumor.

**Conclusions/Significance:**

Our data demonstrate the anti-proliferative and anti-metastatic effects of SAC on HCC cells and suggest that SAC may be a potential therapeutic agent for the treatment of HCC patients.

## Introduction

Hepatocellular carcinoma (HCC) is one of the most life-threatening cancers causing more than half million incidences and deaths per year in the world [1]. Treatment options are greatly limited for patients with advanced HCC due to presence of large size tumors and potential metastasis [2]. Badly, there is no effective systemic chemotherapy for advanced HCC patients. Therefore the development of novel treatment regimens is a pressing need for these patients.

Garlic, a member of *Allium* vegetables, has been applied for medicinal uses a long time ago [3]. *Allium* vegetables derived organosulfur compounds (OSCs) have been found to be potentially preventive and therapeutic agents against cancers [4], [5], [6]. There are two major types of OSCs extracted from garlic: one is lipid soluble type such as diallyl sulfide (DAS), diallyl disulfide (DADS), diallyl trisulfide (DATS) and dithiins, another is water soluble type including S-allylcysteine (SAC) and S-allylmercaptocysteine (SAMC) [6]. Several lines of evidences have proved that SAC is an anti-tumor agent against different human cancers such as prostate cancer [7], [8], breast cancer [9], oral cancer [10], neuroblastoma [11] and non-small-cell lung carcinoma [12]. Moreover, the applications of SAC for *in vivo* treatment of cancers showed no signed toxicity on the nude mice [8]. The major effects of SAC against tumors include induction of apoptosis, inhibition of proliferation and suppression of invasion and adhesion [4], [6], [9], [10], [13]. A recent study indicated that SAC can prevent N-nitrosodiethylamine (NDEA)-induced hepatocarcinogenesis in Wistar rats [14]. Up to now the effect of SAC on treating human HCC has not been studied. We aimed to investigate the potential of SAC in suppressing the proliferation and metastasis of human HCC cells through a series of *in vitro* and *in vivo* experiments. The molecular mechanisms of SAC-induced effects on HCC cells and the possibility of combining SAC with traditional chemotherapy to treat HCC cells were also investigated.

## Materials and Methods

### S-allylcysteine (SAC)

SAC was provided by Wakunaga Pharmaceutical Co., Ltd. (Hiroshima, Japan). A stock solution of SAC (100 mM) was prepared freshly in phosphate-buffering saline (PBS) according to the instruction.

### Cell lines

A human metastatic HCC cell line, MHCC97L, was provided by Liver Cancer Institute & Zhongshan Hospital of Fudan University, Shanghai, China [15]. The cell line was cultured in DMEM high glucose medium (Gibco) with 10% fetal bovine serum (FBS, Gibco) and 1% penicillin and streptomycin in a 37°C incubator supplied with 5% CO_2_. Luciferase gene integrated MHCC97L cells [16], named MHCC97L-Luc, were used for *in vivo* experiment.

### MTT assay

Three thousand cells per well were seeded in 96-well plates and incubated in normal condition for 24 hours. Cells were treated with different concentrations of SAC for 2, 3 and 4 days. Cells were treated with 100 µl of 5 mg/ml of (3-(4,5-Dimethylthiazol-2-yl)-2,5-diphenyltetrazolium bromide (MTT, Invitrogen) solution for 3 hours at 37°C until crystals were formed. MTT solution was removed from each well and 100 µl of DMSO was added to each well to dissolve the formed crystals. Color intensity was measured by Microplate Reader (Model 680, Bio-Rad) at 570 nm. Each experiment consisted of four replications and at least three individual experiments were carried out.

### Colony-forming assay

Cells of single-cell suspension (500 cells per well) were inoculated in 6-well plates and incubated for 24 hours. The cells were treated with different concentrations of SAC for 2 weeks. The cells were fixed by ice-cold methanol and stained by Crystal violet for 10 minutes. Colonies (more than 50 cells) were counted directly on the plate. Statistical significant was calculated from each four independent experiments.

### Wound-healing assay

Cells were seeded onto 24-well plates and incubated for 24 hours to reach 100 percent confluence of the well. A straight-line-wound was made by scraping a 20 µl-pipette tip across the cell monolayer. Cells were rinsed with PBS and cultured with 0 mM, 20 mM and 40 mM of SAC for 48 hours. The wound was captured at 0, 24 and 48 hour under microscopy.

### Invasion assay

Invasion assay was performed by using BD BioCoat™ BD Matrigel™ Invasion Chamber (BD Biosciences). Briefly, 5×10^4^ cells were suspended in 500 µl of serum-free DMEM and seeded into the migration chamber. The migration chamber was placed into a 24-well plate with 500 µl of 0 mM, 20 mM or 40 mM of SAC. After 24 hours of incubation, cells on the upper surface of the chamber were scrapped out by a cotton swab. Cells migrated through the chamber were stained by hematoxylin and eosin (H & E) and subsequently counted under the microscope. At least three independent experiments were performed.

### Apoptosis assay

Cells (3×10^5^) were seeded onto 6-well plate for 24 hours. The cells then were treated with different concentrations of SAC ranging from 0 mM to 40 mM for 2 days. The cells were harvested and stained with Annexin-V-Fluor Staining Kit (Roche) according to manufactory's instruction and analysis by flow cytometer. Early apoptosis was defined as Annexin V-positive and PI-negative cells. Late apoptosis was defined as Annexin V-positive and PI-positive cells. Necrosis was defined as Annexin V-negative and PI-positive cells. Each experiment was analysed in triplicate and at least three independent experiments were performed.

### Cell cycle analysis

Cells (3×10^5^) were seeded onto 6-well plate for 24 hours. The cells were treated with different concentrations of SAC ranging from 0 mM to 40 mM for 24 hours. The cells were trypsinized and fixed with 75% ice-cold ethanol for one hour and stained with 1 µg/ml of propidium iodide (PI) and 0.5 µg/ml of RNase A at 37°C for 30 minutes. DNA content of 10,000 cells from each experiment was analyzed by flow cytometer. Each experiment was analysed in duplicate and four independent experiments were performed.

### Quantitative real-time RT-PCR

The quantitative real-time RT-PCR analysis was conducted as previously described [17]. Briefly, Total RNAs from cells were isolated by TRIZol regent (Invitrogen). cDNA was synthesized by High capacity cDNA Kit (Applied Biosystems). Each of the target genes was amplified by using Power SYBR Green PCR Master Mix (Applied Biosystems) and analyzed by a 7700 Sequence Detection Instrument (Applied Biosystems). Threshold cycle (Ct) value for each sample was determined by the preset parameters of the instrument. Dissociation curve was examined for every PCR reaction to ensure a specific PCR product. Quantitative RT-PCR was performed in triplicates and repeated three times. Primers used including 18S sense 5′-CTCTTAGCTGAGTGTCCCGC-3′; anti-sense 5′-CTGATCGTCTTCGAACCTCC-3′; proliferating cell nuclear antigen (PCNA) sense 5′-GGCTCCATCCTCAAGAAGGT-3′, antisense 5′-CAAAGAGACGTGGGACGAGT-3′; E-cadherin sense 5′-GAACGCATTGCCACATACAC-3′; anti-sense 5′-ATTCGGGCTTGTTGTCATTC-3′; vascular endothelial growth factor (VEGF) sense 5′-CGAAGTGGTGAAGTTCATGGATG-3′, anti-sense 5′-TTCTGTATCAGTCTTTCCTGGT-3′.

### Western blot

Proteins were extracted by 1× Lysis Buffer (Cell Signaling). Protein extracts were separated by 12% SDS-PAGE and transferred to PDMF membrane (Millipore). After blocking with 5% non-fat milk for 1 hour, primary antibody in appropriately dilution was hybridised with the membrane at 4°C over night. The membrane was washed 3 times with TBS/T each for 10 minutes and incubated with secondary antibody for 1 hour at room temperature. Protein signal was detected by ECL Plus system (GE Healthcare). Antibodies used including PCNA, VEGF (Santa Cruz), Caspase-3 (Cell signalling), Cleaved caspase-3 (Cell signalling), Caspase-9 (Cell signalling), Cleaved caspase-9 (Cell signalling), Bcl-2 (Cell signalling), Bcl-xL (Cell signalling), Actin (Santa Cruz), cdc25 (Cell signalling), cdc2 (Cell signalling) and cyclin B1 (Cell signalling).

### Immunofluorescence staining

Cells (3×10^5^) were seeded onto 6-well plate for 24 hours. The cells were treated with different concentrations of SAC ranging from 0 mM to 40 mM for 24 hours. The cells were harvested and fixed with 4% paraformaldehyde at 4°C for 15 min. After washing, the fixed cells were permeabilized by incubated with 0.2% triton X-100 in PBS for 10 min at room temperature. The cells were blocked with 5% BSA for 30 min. The cells were incubated with Ki-67 antibody (BD Biosciences) for 30 min at room temperature. After washing, FITC-conjugated secondary antibody (Santa Cruz) was incubated with the cells for 30 min at room temperature. The labeled cells were analyzed by flow cytometer. Three replicates were performed.

### Orthotopic xenograft nude mice liver tumor model

Male athymic nude mice (BALB/c nu/nu, 4–6 weeks old) were used. The mice were housed in a standard animal laboratory under constant environment conditions with a 12-h light and dark cycle with water and chow. They were kept MHCC97L-luc cells (1×10^6^) in 0.1 ml culture medium were injected subcutaneously into a nude mouse. Once the subcutaneous tumor reached 0.8 to 1 cm in diameter, it was cut into cubes (∼1 to 2 mm^3^ in size) and then implanted into the left liver lobes of nude mice [18]. The treatment started 1-week after the implantation. The mice were divided into three groups: Control (PBS), SAC (1 mg SAC/kg/day) and SAC plus Cisplatin (1 mg SAC/kg/day plus 1 mg cisplatin/kg/day). Xenogen IVIS® *in vivo* imaging system was used to monitor the growth of liver tumor and lung metastasis. The mice were sacrificed at 6-weeks after implantation. Animal studies had been specifically approved by Animal (Control of Experiments) Ordinance Chapter 340, the Department of Health, Hong Kong Special Administrative Region (Ref.: (09–737) in DH/HA&P/8/2/3 Pt. 17).

### Statistical analysis

Statistical analysis was carried out using SPSS 16.0 for Windows (SPSS Inc., IL). For category variables, Chi-square test with Fisher's extract was used. Two-tailed Student's t-test was used for analysis of continuous variables. P<0.05 was considered to be statistically significant.

## Results

### SAC suppresses proliferation of HCC cells

**Figure 1:**
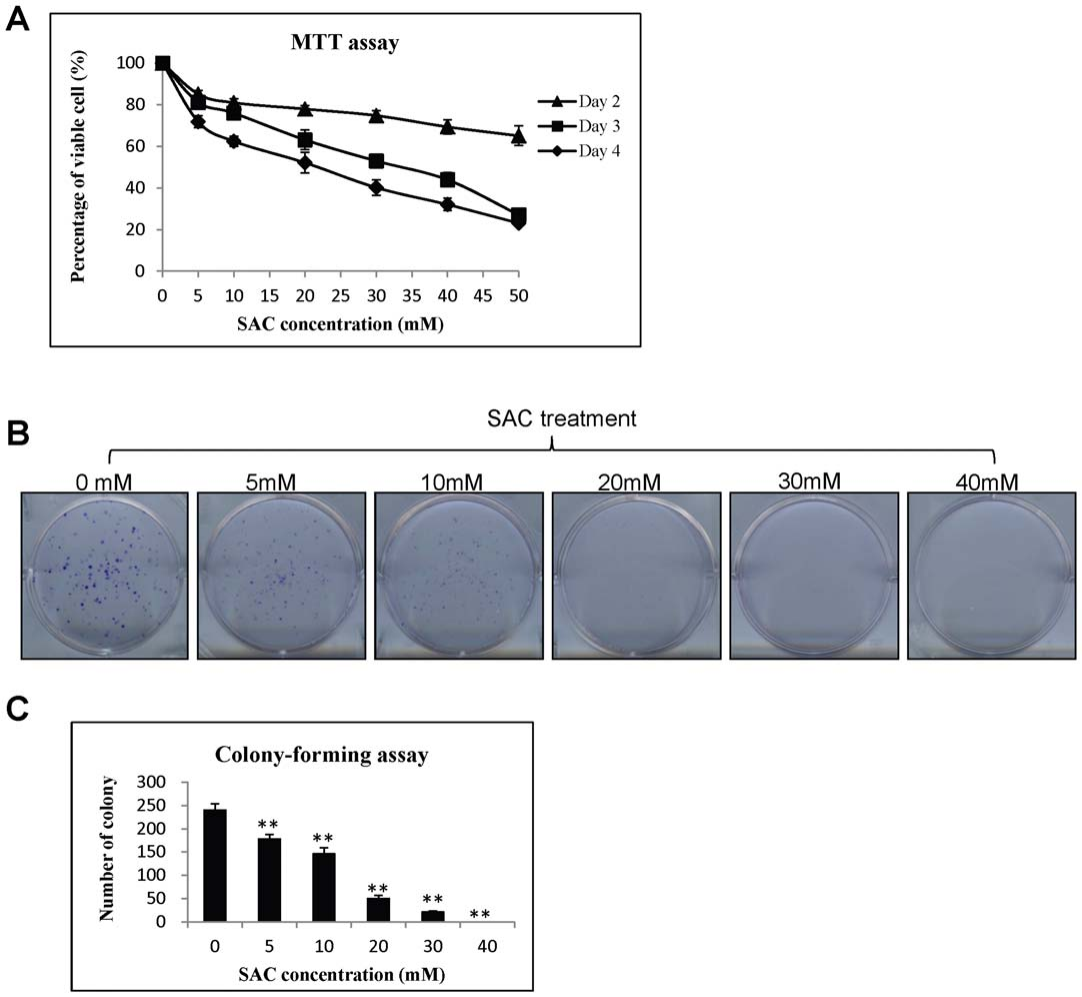
The effect of SAC on proliferation and colony-forming ability of MHCC97L cells. (A) MTT assay at day 2, 3 and 4. (B) Representative plates of colony-forming assay (C) Bar-chart of colony-forming assay. Statistical analysis was performed by comparing to the value of 0 mM SAC treatment. **, p<0.01.

**Figure 2:**
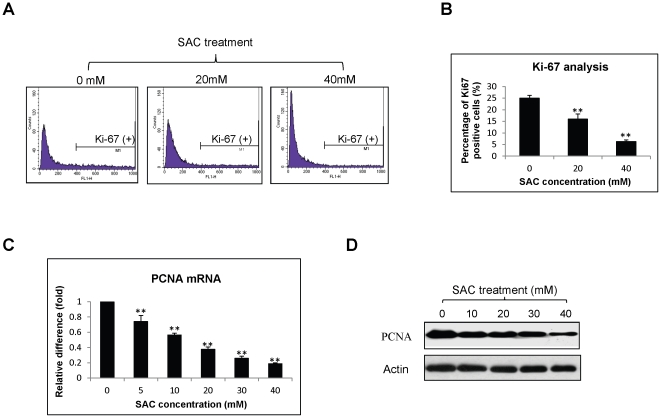
The effect of SAC on the expressions of Ki-67 and PCNA of MHCC97L cells. (A) Representative immunofluorescent analysis of Ki-67 protein after SAC treatment for 48 hours. X-axis represents Ki-67 intensity and Y-axis represents cell number. (B) Bar-chart of immunofluorescent analysis of Ki67 protein under SAC treatment. (C) Quantitative real-time RT-PCR and (D) Western blot analyses of PCNA mRNA and protein respectively after SAC treatment for 48 hours. Statistical analysis was performed by comparing to the value of 0 mM SAC treatment. **, P<0.01.

To investigate the effect of SAC on the growth of HCC cells, MTT and colony-forming assays were performed. MTT assay demonstrated that SAC suppressed the proliferation of MHCC97L cells in time- and dose-dependent manners (Figure 1A). The highest concentration of SAC did not reach the half maximal inhibitory concentration (IC_50_) to MHCC97L cells at day 2. SAC exhibited an increasing suppressive effect on MHCC97L cells after 3 days of treatment. The IC_50_ of SAC to MHCC97L cells was about 33 mM and 22 mM at day 3 and day 4 respectively. Moreover, colony-forming ability of MHCC97L cells was significantly inhibited by SAC treatment with concentrations from 5 mM to 40 mM (Figure 1B and 1C). There was no colony formed in 40 mM of SAC. To elucidate the anti-proliferative mechanism of SAC on MHCC97L cells, the expressions of two proliferative markers, Ki-67 and PCNA, were examined. The study of immunofluorescent staining of Ki-67 antibody showed that the number of Ki-67 stained MHCC97L cells was significantly reduced after treatment with 20 mM and 40 mM of SAC for 48 hours (Fig. 2A and 2B). There was a more than 4-folds down-regulation of Ki-67 expression in MHCC97L cells after 40 mM SAC treatment compared with the cells without SAC treatment (6.3% *vs* 25.0%, Fig. 2B). Additionally, the expressions of PCNA mRNA and protein were significantly down-regulated after 48-hour SAC treatment in a dose-dependent manner (Fig. 2C and 2D).

### SAC induces apoptosis of HCC cells

**Figure 3:**
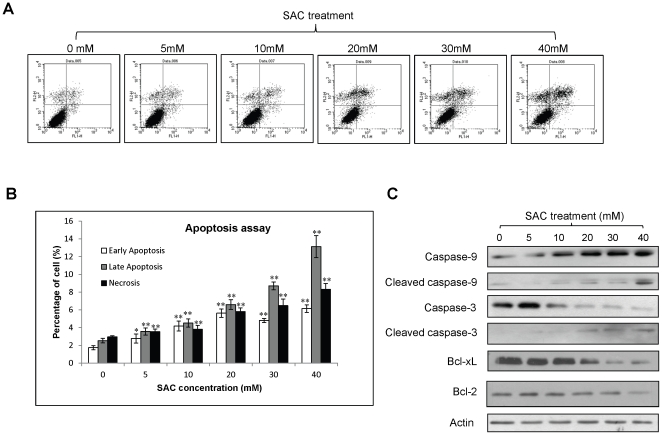
The effect of SAC of apoptosis of MHCC97L cells. (A) Flow cytometry analysis of MHCC97L cells. X-axis represents Annexin V label and Y-axis represents PI label. (B) Summary of apoptosis assay. (C) Western blot analysis of apoptosis proteins under SAC treatment. Statistical analysis was performed by comparing to the value of 0 mM SAC treatment. *, p<0.05; **, P<0.01.

Anti-apoptosis is one of the important mechanisms that HCC cells utilize it to protect themselves from chemotherapies. To investigate whether SAC affects apoptosis in HCC cells, Annexin V and PI staining was performed. The result of apoptosis assay demonstrated that the percentages of the early apoptotic, late apoptotic and necrotic cells of MHCC97L cells were significantly increased after treatments with SAC for 48 hours in a dose-dependent manner (Fig. 3A and 3B). Among them, late apoptosis was the most influenced one by SAC. There were 3.5-, 5.2- and 2.8-fold increases of early apoptosis, late apoptosis and necrosis of MHCC97L cells respectively at 40 mM SAC treatment compared with the cells without treatment (Fig. 3B). By combining the percentages of early and late apoptotic cells, SAC treatment at 40 mM caused a 4.5-fold increase of the total percentage of apoptotic cells compared to the cells without treatment. Western blot analysis showed that cleaved caspase-3 and cleaved caspase-9 of MHCC97L cells were activated along with SAC treatment and both reached the highest level at 40 mM of SAC (Fig. 3C). The protein level of caspase-9 was increased along with SAC treatment while the protein level of caspase-3 was decreased (Fig. 3C). Moreover, the expressions of anti-apoptotic proteins Bcl-xL and Bcl-2 in MHCC97L cells were suppressed by SAC treatment (Fig. 3C). The suppressive effect of SAC on Bcl-xL was found to be a dose-dependent manner and was more obvious than on Bcl-2 (Fig. 3C).

### SAC arrests S phase of HCC cells

**Figure 4:**
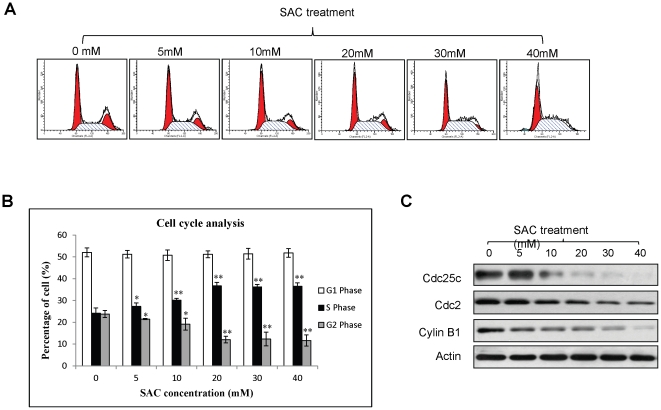
The effect of SAC of cell cycle of MHCC97L cells. (A) Representative histograms of cell cycle analysis. Y-axis represent number of cells; X-axis represents DNA content (PI intensity). (B) Distribution of G2, S and G1 phases of MHCC97L cells under SAC treatment. (C) Western blot analysis of S phase regulated proteins. Statistical analysis was performed by comparing to the value of 0 mM SAC treatment. *, p<0.05; **, P<0.01.

To investigate the effect of SAC on cell cycle of HCC, DNA content of MHCC97L cells was analyzed by PI staining. Our result showed that SAC treatment resulted in a significant increase of S phase content of MHCC97L cells (Fig. 4A and 4B). The content of S phase of MHCC97L cells was increased to more than 36% after ≤20 mM of SAC was administrated while the content of S phase of the cells without treatment was 24% (Fig. 4B). The significant arrest in S phase caused a significant decrease of G2/M phase content (Fig. 4B). There was no significant change of G1 phase between SAC treated and non-treated MHCC97L cells (Fig. 4B). Western blot analysis showed that the expressions of cdc25c, cdc2 and cyclin B1 proteins in MHCC97L cells were suppressed after SAC treatment in a dose-dependent manner (Fig. 4C).

### SAC suppresses migration and invasion of HCC cells

**Figure 5:**
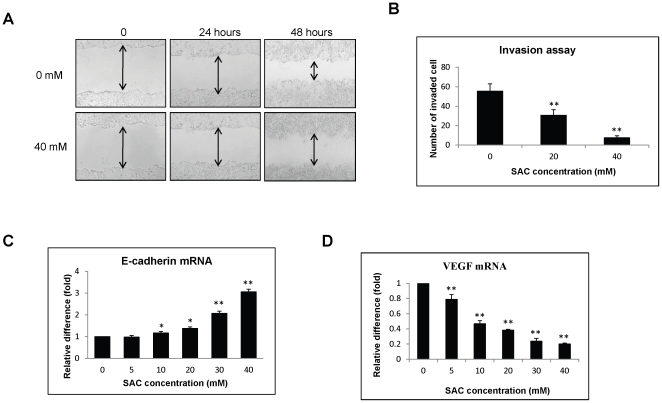
The effect of SAC on migration and invasion of MHCC97L cells. (A) Wound-healing assay of MHCC97L cells under SAC treatment. (B)Invasion assay. Quantitative real-time RT-PCR analyses of (C) E-cadherin and (D) VEGF mRNA. Statistical analysis was performed by comparing to the value of 0 mM SAC treatment. *, p<0.05; **, P<0.01.

To further investigate the anti-metastatic property of SAC on HCC cells, wound healing assay and invasion assay were performed to study the changes of *in vitro* migration and invasion abilities of MHCC97L cells under SAC treatment. Wound-healing assay demonstrated that the migration ability of MHCC97L cells after creation of the wound was hindered by treatment of 40 mM SAC compared to the cells without SAC treatment (Fig. 5A). Moreover, administration of SAC at the concentration of 20 mM and 40 mM significantly inhibited the invasion ability of MHCC97L (Fig. 5B). To understand the molecular mechanism of the effect of SAC on the metastatic ability of HCC cells, the expressions of two important metastasis-related genes including E-cadherin and VEGF were analyzed by quantitative real-time RT-PCR. Significant and dose-dependent up-regulation of E-cadherin mRNA (Fig. 5C) and down-regulation of VEGF mRNA (Fig. 3D) were detected in MHCC97L cells after treatment of SAC.

### SAC suppresses *in vivo* growth and metastasis of HCC cells

**Figure 6:**
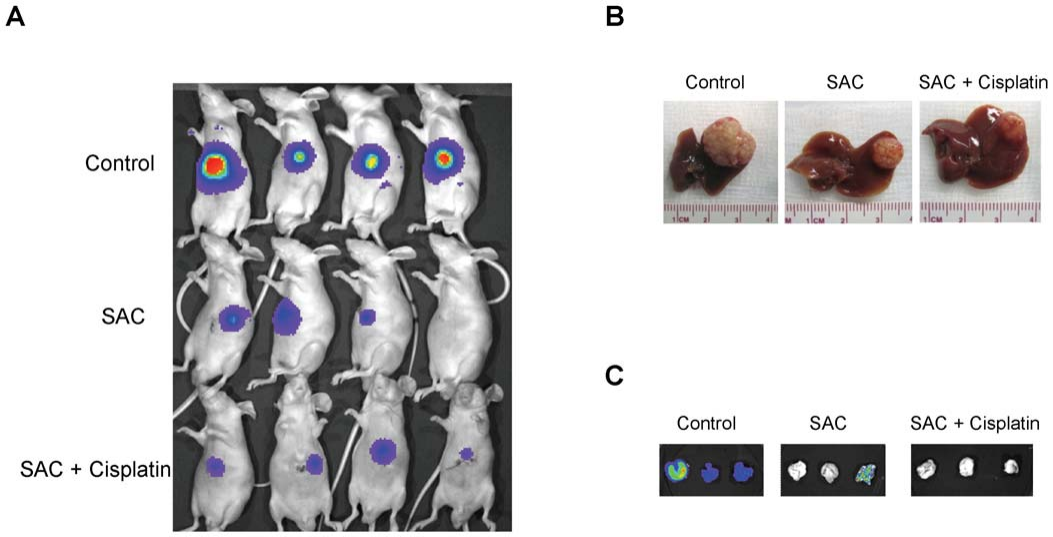
The Effect of SAC of *in vivo* tumor growth and metastasis of MHCC97L cells. (A) *In vivo* images of rats by Xenogen IVIS® imaging system at week 6 after implantation. (B) Tumor-bearing liver tissues (C) *In vivo* images of lung tissues by Xenogen IVIS® imaging system.

Single SAC treatment and SAC combined with cisplatin treatment significantly reduced the *in vivo* tumor volume of MHCC97L-Luc cells to approximately 4 and 6 folds respectively (Fig. 6A, 6B and Table 1). There was no significantly difference between single and combination treatment on suppressing tumor growth (Table 1). The lung metastatic potential of MHCC97L-Luc was suppressed by SAC single or combined with cisplatin treatment (Fig. 6C). Single SAC treatment reduced the lung metastatic rate of MHCC97L cells from 87.5% to 37.5%, but there was no statistical significance between single SAC treatment group and control group (Table 1). SAC combined with cisplatin significantly reduced the lung metastatic rate of MHCC97L-Luc cells to 12.5% compared to the control group (Table 1). There was no statistically significant difference between single and combination treatments in suppressing lung metastasis of HCC cells (Table 1).

**Table 1:** Summary of orthotopic xenograft liver tumor model.

	Control (n = 8)	SAC (n = 8)	SAC+Cisplatin (n = 8)	*P* value (SAC *vs* Control)	*P* value (SAC+cisplatin *vs* Control )	*P* value (SAC+cisplatin *vs* SAC)
Tumor formed	8/8(100%)	8/8(100%)	7/8(87.5%)	NS	NS	NS
Tumor volume (cm^3^)	2.02±0.51	0.53±0.26	0.32±0.14	0.00	0.00	NS
Pulmonary metastasis	7/8(87.5%)	3/8(37.5%)	1/8(12.5%)	NS	0.01	NS

NS = not significant.

## Discussion

Surgical treatments such as liver resection and liver transplantation provide better clinical outcome for patients with early stage of HCC. Up to 70% of HCC patients have been suffering from limited treatment options due to late diagnosis and/or advanced stage of the disease when however, surgical treatments as well as regional therapy are not feasible. So far, targeted therapy is no even satisfactory for advanced HCC patients [2]. Therefore, we aimed to seek for alternative regimens which can effectively eradicate the progression and metastasis of HCC. SAC, a garlic derived water soluble organosulfur compound (OSC), has been demonstrated to have anti-tumor effects in different human cancers [7], [9], [10], [11], [12], [13]. With no previous report of SAC in the treatment of human HCC, we employed a series of *in vitro* and *in vivo* functional studies to investigate the anti-proliferative and anti-metastatic effects of this agent on a human metastatic HCC cell line.

In this study, we demonstrated that SAC could inhibit the proliferation rate of MHCC97L cells in dose- and time-dependent manners. The anti-proliferative effect of SAC on MHCC97L cells was more notable after more than 3 days of treatment. These phenomena were comparable to the effect of SAC on other cancers such as breast, prostate and lung cancers [7], [9], [12], suggesting a common effective action time of SAC on cancers. Moreover, SAC significantly inhibited the colony-forming ability of HCC97L cells from single cell, indicating its suppressive effect on HCC initiation. Furthermore, the animal study demonstrated that administration of SAC significantly inhibited the *in vivo* growth of MHCC97L-Luc cells. The anti-proliferative effect of SAC on MHCC97L cells was explained to be its suppressive effect on the expressions of proliferative markers including Ki-67 and PCNA. High label index of PCNA has been found to be significantly associated with larger tumor size, high recurrence rate and poor survival outcome of HCC patients after hepatectomy [19]. The anti-proliferative effect of SAC on HCC cells through suppression of PCNA indicates it possible therapeutic application for treatment of HCC patients.

Evasion of apoptosis is one of the hallmarks of cancers [20]. Members of *Allium* vegetable-derived OSCs have been found to suppress the growth of cancers through induction of apoptosis of tumor cells [5], [6]. Currently, the effect of SAC on apoptosis of cancers has been elusive. In human prostate cancer, SAC can elevate apoptosis of the cancer cells under *in vivo* environment through activation of cleaved caspase-3 and down-regulation of Bcl-2 [8]. In this study, we found that SAC could significantly induce apoptosis and necrosis of HCC cells in a dose-dependent manner. Moreover, SAC treatment on HCC cells could lead to activations of cleaved caspase-3 and cleaved caspase-9 as well as down-regulation of Bcl-xL and Bcl-2 expressions. Bcl-xL and Bcl-2 which have anti-apoptotic function by protecting mitochondria from cytochrome c release are commonly over-expressed in cancers [20]. Therefore, these results suggested that the suppressive effect of SAC of HCC cells might be attributed to the induction of caspase-mediated apoptosis through down-regulation of anti-apoptotic proteins.

Several lines of evidences suggest that cancer cells treated with OSCs can lead to the arrest in G2/M phase of the cell cycle through modulating the expressions or activities of cyclins, cyclin-dependent kinases (Cdks) , signaling molecules and histones [6]. A study showed that SAMC but not SAC inhibits the growth of human colon cancer cell lines at G2/M phase of the cell cycle [21]. In this study, we demonstrated, for the first time, that SAC caused a significant accumulation of MHCC97L cells at S phase resulting in significantly decreased number of cells at G2/M phase of the cell cycle, suggesting that SAC might suppress the proliferation of HCC cells by inducing cell cycle at S/G2 transition. Inhibition of cdc25c, cdc2 and cyclin B1 is associated with the delays of S phase progression of HCC cells after interferon-α treatment [22]. Our result showed that the expressions of cdc25c, cdc2 and cyclin B1 in HCC cells were down-regulated by SAC treatment in a dose-dependent manner, indicating SAC may delay S phase progression to G2/M phase of HCC cells through suppression of cell cycle regulators. Moreover, cdc25c, cdc2 and cyclin B1 are important regulators in controlling G2/M transition of the cell cycle [23]. Arrest in G2/M phase of cancer cells by other OSCs, such as DADS, DAS and DATS, has been found to be associated with down-regulation of cdc25c proteins [6], [24]. Thus, down-regulation of cdc25c, cdc2 and cyclin B1 by SAC might also suggest the influence of SAC on G2/M transition of HCC cells. Cdc2 and cyclin B1 are commonly overexpressed in HCC patients and the overexpressions of these genes are positively associated with higher malignant status and poor prognosis of HCC patients [25], [26]. Suppression of cdc2 and cyclin B1 expressions by SAC might suggest the therapeutic implication of SAC for the treatment of HCC patients.

Patients with advanced or metastatic HCC only rely on systemic chemotherapy which cannot achieve improved overall survival for these patients [2], [27]. Apart from anti-proliferative effect on cancers, SAC has been found to inhibit the invasion of cancer cells such as breast and prostate cancer cells by modulating the expression of E-cadherin [7], [9]. Therefore, the effect of SAC on HCC metastasis was also investigated in this study. MHCC97L is a metastatic cell line which can metastasize to the lung from the liver [16], [28]. Our *in vitro* study demonstrated that SAC effectively suppressed the migration and invasion abilities of MHCC97L cells. Furthermore, *in vivo* study showed that SAC could reduce the metastatic potential of MHCC97L-Luc cells from 87.5% to 37.5%, suggesting an anti-metastatic effect of SAC on HCC metastasis. Moreover, combination of SAC and cisplatin could significantly inhibit lung metastasis of MHCC97L-luc cells, indicating its synergetic implication on HCC treatment. Metastasis is a multi-step process which is composed of invasion, intravasion, arrest in bloodstream, extravasion and metastatic colonization [29]. E-cadherin and VEGF are two important regulators of cancer metastasis [29]. The decrease or loss of E-cadherin has been frequently found in tumor tissues of HCC patients which is significantly associated with advanced HCC stages and high recurrent rate after liver resection [30]. Up-regulation of E-cadherin by TGF-beta inhibitor can hinder the epithelial-mesenchymal transition (EMT) process of HCC cells resulting in suppression of migration and invasion [31]. VEGF is a critical factor of angiogenesis whose up-regulation in HCC patients is significantly associated with high proliferative index, angiogenesis, tumor invasion and poor prognosis after liver resection [32]. Targeted inhibition of VEGF has been proved to improve clinical outcome of advanced HCC patients [33]. In this study, SAC could restore the expression of E-cadherin and suppress the expression of VEGF of MHCC97L cells, suggesting its anti-metastatic effect on HCC through inhibition of HCC invasion and angiogenesis.

In summary, our data demonstrated that SAC could suppress the proliferation and metastatic potential of HCC by modulating important regulators involved in proliferation, invasion, apoptosis, cell cycle and angiogenesis, suggesting that SAC may be a potential therapeutic agent for the treatment of HCC patients.
